# Anion Gap Was Associated with Inhospital Mortality and Adverse Clinical Outcomes of Coronary Care Unit Patients

**DOI:** 10.1155/2020/4598462

**Published:** 2020-08-27

**Authors:** Tienan Sun, Chenghui Cai, Hua Shen, Jiaqi Yang, Qianyun Guo, Jingrui Zhang, Biyang Zhang, Yaodong Ding, Yujie Zhou

**Affiliations:** ^1^Beijing Anzhen Hospital Affiliated to Capital Medical University, Beijing, China; ^2^Beijing Institute of Heart, Lung and Blood Vessel Disease, Beijing, China

## Abstract

**Background:**

Anion gap (AG) has been proved to be associated with prognosis of many cardiovascular diseases. This study is aimed at exploring the association of AG with inhospital all-cause mortality and adverse clinical outcomes in coronary care unit (CCU) patients.

**Method:**

All data of this study was extracted from Medical Information Mart for Intensive Care III (MIMIC-III, version 1.4) database. All patients were divided into four groups according to AG quartiles. Primary outcome was inhospital all-cause mortality. Lowess smoothing curve was drawn to describe the overall trend of inhospital mortality. Binary logistic regression analysis was performed to determine the independent effect of AG on inhospital mortality.

**Result:**

A total of 3593 patients were enrolled in this study. In unadjusted model, as AG quartiles increased, inhospital mortality increased significantly, OR increased stepwise from quartile 2 (OR, 95% CI: 1.01, 0.74-1.38, *P* = 0.958) to quartile 4 (OR, 95% CI: 2.72, 2.08-3.55, *P* < 0.001). After adjusting for possible confounding variables, this association was attenuated, but still remained statistically significant (quartile 1 vs. quartile 4: OR, 95% CI: 1.02, 0.72-1.45 vs. 1.49, 1.07-2.09, *P* = 0.019). Moreover, CCU mortality (*P* < 0.001) and rate of acute kidney injury (*P* < 0.001) were proved to be higher in the highest AG quartiles. Length of CCU (*P* < 0.001) and hospital stay (*P* < 0.001) prolonged significantly in higher AG quartiles. Maximum sequential organ failure assessment score (SOFA) (*P* < 0.001) and simplified acute physiology score II (SAPSII) (*P* < 0.001) increased significantly as AG quartiles increased. Moderate predictive ability of AG on inhospital (AUC: 0.6291), CCU mortality (AUC: 0.6355), and acute kidney injury (AUC: 0.6096) was confirmed. The interactions were proved to be significant in hypercholesterolemia, congestive heart failure, chronic lung disease, respiratory failure, oral anticoagulants, Beta-blocks, angiotensin-converting enzyme inhibitor (ACEI)/angiotensin receptor blocker (ARB), and vasopressin treatment subgroups.

**Conclusion:**

AG was an independent risk factor of inhospital all-cause mortality and was associated with adverse clinical outcomes in CCU patients.

## 1. Introduction

Despite extraordinary progress in cardiovascular field in recent decades, cardiovascular diseases still remain the major cause of death all over the world, causing about 17.5 million deaths each year [1, 2]. Originating in 1962, coronary care unit (CCU) focuses on the treatment of patients with severe cardiovascular diseases, which greatly reduces the mortality rate of patients [3–7]. A cheap and readily available clinical indicator for assessing prognosis still makes sense for CCU patients.

As a traditional clinical indicator used to evaluate acid-base balance, anion gap (AG) has been used in clinical practice for more than 50 years [8, 9]. AG has been proved to be associated with prognosis of many cardiovascular diseases [10–14]. A meta-analysis proved that AG was strongly related to mortality in critically ill patients [15]. Moreover, AG was confirmed to be associated with higher blood pressure [16], insulin resistance [17], and cardiorespiratory fitness [18]. In general population, higher AG was also proved to be related to cardiovascular mortality [19]. On the basis of these evidence, we hypothesized that AG could influence the prognosis of CCU patients. The purpose of this study was to explore the relationship between AG and outcomes of CCU patients.

## 2. Method

### 2.1. Data Source

We retrieved all data from an openly available critical care database named Medical Information Mart for Intensive Care III (MIMIC-III, version 1.4) [20], which included more than 60000 intensive care unit (ICU) stays and more than 50000 stays for adult patients. The data in MIMIC-III were collected from June 2001 to October 2012 in Beth Israel Deaconess Medical Center, including general information (patient demographics, birth and death, ICU admission, and discharge information), vital signs, laboratory data, the balance of body fluid, reports, medication, and nursing record. Protecting Human Research Participant exam was passed to gain access to MIMIC-III database, and our certificate number is 9027152. Structured Query Language (SQL) was used to extract all patient information from MIMIC-III database.

### 2.2. Study Population

**Figure 1:**
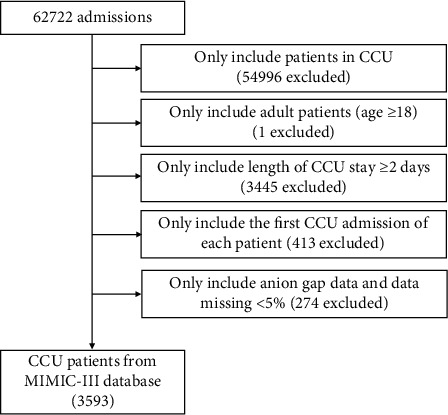
Flowchart of study population. CCU: coronary care unit.

All adult patients (≥18 years) admitted to CCU from MIMIC-III database were included. And only the first admission of each patient was included. Patients meeting the following criteria were excluded: (1) patients were under 18 years old, (2) length of CCU stay <2 days, (3) anion gap data missing, and (4) individual data missing ≥5%. A total of 3593 patients were included in this study (Figure 1).

### 2.3. Data Collection

All data used in this study was extracted using SQL from MIMIC-III database. Demographics, diagnoses of heart diseases, comorbidities and medical history, laboratory parameters, medication use, and survival data were collected. Demographic data included age, gender, and race. Diagnoses of heart diseases included coronary artery disease, acute myocardial infarction, third-degree atrioventricular block, atrial fibrillation, congestive heart failure, ventricular arrhythmias (ventricular tachycardia, ventricular flutter, and ventricular fibrillation), primary cardiomyopathy (hypertrophic obstructive cardiomyopathy and other primary cardiomyopathies), valve diseases (disorders of mitral, aortic, pulmonary, and tricuspid valve; rheumatic diseases of valves and congenital diseases of valves), endocarditis, and cardiogenic shock. Cardiogenic shock was identified by the presence of appropriate International Classification of Diseases, Ninth Versions (ICD-9) diagnosis and procedure code, which was adopted by the World Health Organization to code diagnoses, and previous study confirmed the validity of ICD-9 code in recording clinical conditions in dually coded database [21]. The ICD-9 code of cardiogenic shock used in this study was 78551. Comorbidities and medical history included hypertension, diabetes, chronic liver disease, hypercholesterolemia, chronic lung disease, chronic kidney disease, malignancy, autoimmune diseases, respiratory failure, prior myocardial infarction, and prior stroke. Medication use included antiplatelet, oral anticoagulant, Beta-blocks, ACEI, ARB, statin, and vasopressin. Laboratory parameters included AG, white blood cell, platelet, hemoglobin, creatinine, blood nitrogen urea, sodium, potassium, and glucose. All laboratory parameters were extracted within 24 hours after admission to CCU.

AG was calculated by the following formula: [AG = Na^+^(mmol/L) + K^+^(mmol/L)] − [Cl^−^(mmol/L) + HCO3^−^(mmol/L)], which was generally acknowledged [22]. And AG was recorded as initial AG and maximum AG, initial AG was the first test value after admission to CCU, and maximum AG was the maximum value during CCU stay.

### 2.4. Outcomes

The primary outcome was inhospital all-cause mortality, secondary outcomes included CCU all-cause mortality, acute kidney injury, maximum SOFA [23], maximum SAPSII [24], length of CCU, and hospital stay. Kidney Disease: Improving Global Outcomes (KDIGO) definition [25] was used for diagnosis of acute kidney injury.

Survival information was extracted from table named “patients” of MIMIC-III database. Data of length of CCU stay was extracted from table named “icustays” of MIMIC-III database. Data of length of hospital stay was extracted from table named “admissions” of MIMIC-III database. Acute kidney injury was confirmed based on KDIGO definition from table named “kdigo_creat” and “kdigo_uo” of MIMIC-III database. SOFA was extracted from table named “sofa” of MIMIC-III database. SAPSII was extracted from table named “sapsii” of MIMIC-III database.

### 2.5. Statistical Analysis

All the patients were stratified by AG quartiles. Continuous variables were summarized as mean ± standard deviation (SD) and median [interquartile range (IQR)]. Kruskal–Wallis test or one-way ANOVA analysis was used to test for difference. Categorical variables were summarized as number (percentage) and compared between groups using Chi-square test.

Binary logistic regression analysis was applied to identify the association between AG and inhospital all-cause mortality, and results were summarized as odds ratio (OR) with 95% confidence interval (CI). Covariates were incorporated into regression models based on statistical evidence and clinical judgment. Local weighted regression (Lowess) was applied to fit out curves in line with overall trend. Relative operating characteristic (ROC) curves were used to evaluate predictive ability of AG on inhospital all-cause mortality. All data processing and analysis were performed by Stata V.11.2. All tests were two sided, and *P* < 0.05 was considered statistically significant.

## 3. Result

### 3.1. Patient Characteristics

After screening step by step, a total of 3593 patients admitted to CCU were extracted from MIMIC-III database (Figure 1), most of whom were white and male. The baseline characteristics of patients stratified by AG quartiles are presented in Table 1. Initial AG and maximum AG of all patients were 15.0 ± 3.6 mmol/L and 17.7 ± 4.3 mmol/L, respectively. As AG quartiles increased, rates of ventricular arrhythmias, congestive heart failure, primary cardiomyopathy, cardiogenic shock, diabetes, respiratory failure, and chronic kidney diseases increased. But rates of coronary artery disease, hypertension, hypercholesterolemia, chronic lung diseases, and malignancy decreased as AG quartiles increased. Moreover, patients with higher AG had higher white blood cell, platelet, hemoglobin, glucose, creatinine, blood nitrogen urea, and potassium. Patients with higher AG also received less ACEI/ARB and statin treatment but more vasopressin treatment.

### 3.2. Outcomes

**Figure 2:**
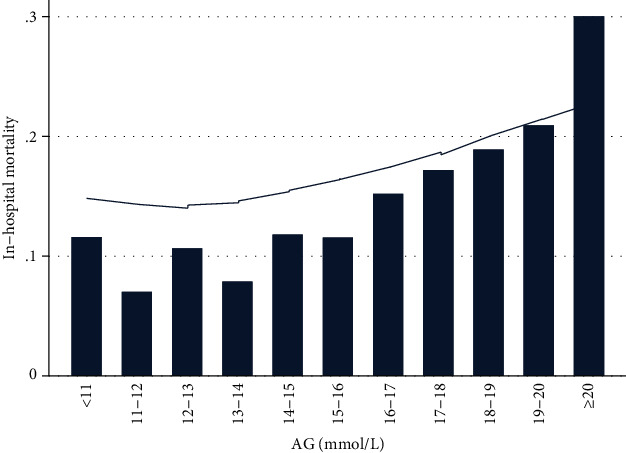
Association between anion gap and inhospital all-cause mortality presented through Lowess smoothing. Abbreviation: AG: anion gap.

The primary outcome was inhospital all-cause mortality. As shown in Table 2, the inhospital mortality of all patients in this study was 14.3%. As AG quartiles increased, inhospital mortality increased gradually (quartile 1 vs. quartile 4: 9.8% vs. 22.8%, *P* < 0.001); the same conclusion was drawn by Lowess smoothing curve shown in Figure 2. From unadjusted model comparing inhospital all-cause mortality among different AG groups, we observed that as AG quartiles increased, inhospital mortality increased significantly, OR increased stepwise from quartile 2 (OR, 95% CI: 1.01, 0.74-1.38, *P* = 0.958) to quartile 4 (OR, 95% CI: 2.72, 2.08-3.55, *P* < 0.001). After adjusting for more variables in model 3, this association was weakened, but still remained statistically significant (quartile 1 vs. quartile 4: OR, 95% CI: 1.02, 0.72-1.45 vs. 1.49, 1.07-2.09, *P* = 0.019) (Table 3).

Secondary outcomes were CCU mortality, acute kidney injury, maximum SOFA, maximum SAPSII, length of CCU, and hospital stay. As shown in Table 2, CCU mortality of all patients was 11.1%, and as AG quartiles increased, the rate of CCU mortality increased stepwise from quartile 1 (6.3%) to quartile 4 (17.5%) (*P* < 0.001). Length of CCU stay (quartile 1 vs. quartile 4: 3.5 (2.6-5.9) vs. 4.4 (3.0-7.3), *P* < 0.001) and length of hospital staying (quartile 1 vs. quartile 4: 7.3 (4.8-12.5) vs. 9.7 (4.0-15.7), *P* < 0.001) increased significantly as AG increased. A total of 1924 patients were diagnosed with acute kidney injury based on KDIGO definition, and the incidence of acute kidney injury increased gradually from quartile 1 (42.7%) to quartile 4 (66.1%) (*P* < 0.001). Moreover, patients in highest AG quartile had highest maximum SAPSII (quartile 1 vs. quartile 4: 33 (27-40) vs. 42 (33-52), *P* < 0.001) and highest maximum SOFA (quartile 1 vs. quartile 4: 3 (1-5) vs. 5 (3-8), *P* < 0.001).

The relationship between inhospital mortality and AG quartiles in different subgroups is shown in Table 4. We did not observe significant interactions in most subgroups. Patients with chronic lung disease, congestive heart failure, and respiratory failure had lower risk of inhospital death. Moreover, patients who received oral anticoagulants, Beta-blocks, ACEI/ARB, and vasopressin treatment had lower risk of inhospital death too. But patients with hypercholesterolemia had higher risk of inhospital death.

**Figure 3:**
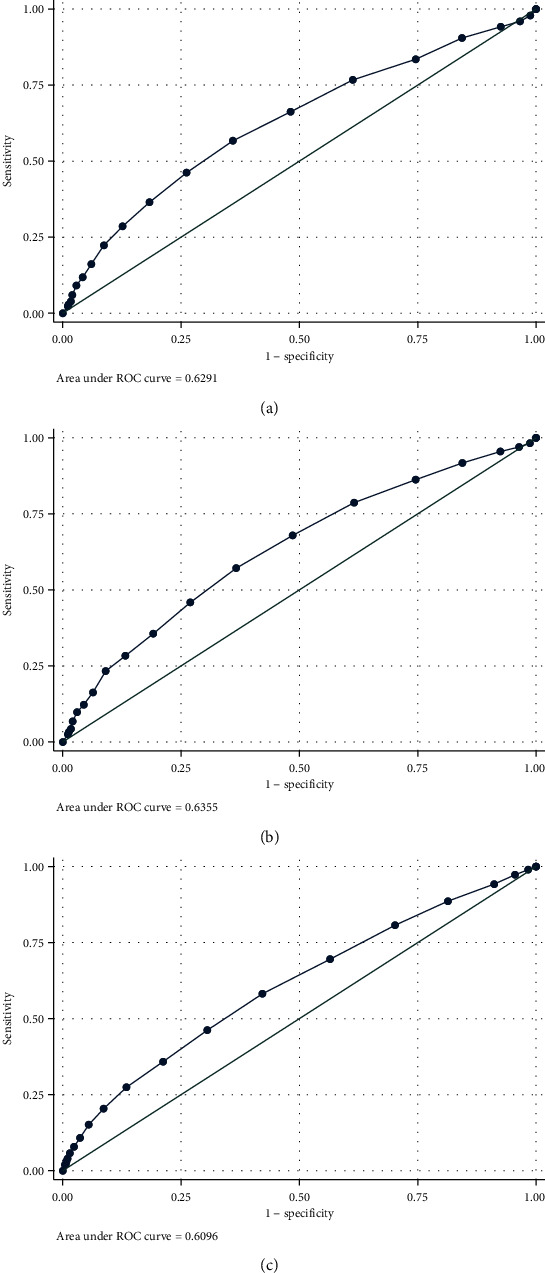
ROC curves of AG for prediction of inhospital all-cause mortality (a), CCU all-cause mortality (b), and acute kidney injury (c). Abbreviation: AG: anion gap; CCU: coronary care unit.

As presented in Figure 3, moderate predictive ability of AG on inhospital (AUC, 95% CI: 0.6291, 0.6019-0.6564), CCU mortality (AUC, 95% CI: 0.6355, 0.6060-0.6650), and acute kidney injury (AUC, 95% CI: 0.6096, 0.5914-0.6278) was confirmed.

## 4. Discussion

This study explored the association of AG with inhospital mortality and other adverse outcomes of CCU patients. The main findings were as follows: (1) as AG quartiles increased, inhospital all-cause mortality increased significantly. (2) As AG quartiles increased, CCU mortality and the rate of acute kidney injury increased. (3) Patients with higher AG had higher maximum SOFA and maximum SAPSII. (4) Length of CCU and hospital stay prolonged significantly in higher AG quartiles. (5) Moderate predictive ability of AG on inhospital, CCU mortality, and acute kidney injury was confirmed. (6) The interactions were proved to be significant in hypercholesterolemia, congestive heart failure, chronic lung disease, respiratory failure, oral anticoagulants, Beta-blocks, ACEI/ARB, and vasopressin treatment subgroups.

Acid-base balance is very important for the maintenance of normal physiological function and cell metabolism [26]. As a common indicator to evaluate acid-base balance, AG is often used to define the types and causes of metabolic acidosis. Clinically, AG is usually calculated by the concentration of serum sodium, potassium, chloride, and bicarbonate [27], which is inexpensive and readily available.

AG has been proved to be associated with prognosis of many cardiovascular diseases [10–14]. Previous study which enrolled 18115 patients with coronary disease showed that higher AG was associated with worse cardiac function, more severe clinical symptoms, and acute myocardial infarction: for every unit increase in AG, the 30-day risk of all-cause death increased by 0.244 times [10]. In patients with myocardial infarction, increased AG was also associated with higher mortality and cardiogenic shock [12]. Another research enrolled 63 patients with cardiogenic shock following ST-segment elevation myocardial infarction (STEMI) came to a similar conclusion that higher AG was associated with higher mortality [13]. For patients with STEMI, AG was proved to be an independent risk factor for high inhospital mortality after percutaneous coronary intervention and could be used for risk stratification [14]. Moreover, a meta-analysis and another study revealed that AG may be a good choice to assess the prognosis of critically ill patients especially for those in areas with inadequate medical resources [15, 28]. Similarly, our data suggested that AG was associated with inhospital all-cause mortality of CCU patients independently, and maybe the adverse effects of increased AG on coronary artery disease and critically ill patients contribute to this result. Moreover, AG contributed to the diagnosis of acute kidney injury [8, 27]. Similarly, we found that as AG quartiles increased, incidence of acute kidney injury increased significantly. SOFA and SAPSII were good scoring system for predicting the prognosis of critically ill patients. In this study, we found that as AG increased, SOFA and SAPSII increased significantly, and this phenomenon may also explain higher inhospital mortality in patients with higher AG. Moreover, length of CCU and hospital stay prolonged significantly in higher AG quartiles, which will bring greater psychological, physical, and financial burden to patients, so more attention to AG in CCU patients may be needed.

In coronary artery disease, acute myocardial infarction, third-degree atrioventricular block, congestive heart failure, primary cardiomyopathy, valve disease, ventricular arrhythmias, endocarditis, cardiogenic shock, and atrial fibrillation subgroups, we can all come to the same conclusion that as AG increased, inhospital mortality increased. All above diseases almost covered most diseases of CCU. This result provided a strong support for us to use AG as a clinical indicator in CCU to predict prognosis. In hypercholesterolemia, congestive heart failure, chronic lung disease, respiratory failure, oral anticoagulants, Beta-blocks, ACEI/ARB, and vasopressin subgroups, the interactions were proved to be significant. Further research is needed to clarify the reasons.

## 5. Limitation

This study was a single retrospective study, and inevitable bias may affect the authenticity of the results. Moreover, the bulk of AG is largely determined by anions attached to circulation protein [29, 30], and as albumin decreases, so does AG [31, 32]. But due to the loss of albumin data, we did not include the albumin data in this study. Apart from the retrospective model, the main bias of this study was the lack of albumin values for a correct AG adjustment. In general, the more key variables a model contains, the more accurate its predictions will be. But constrained by public databases, a lot of information that may affect the model was not collected, like smoking and drinking alcohol. In addition to this, other important information was also not collected such as specific cause of death, cardiac function, and left ventricular ejection fraction. In order to verify the conclusion, prospective case-control study may be needed.

## 6. Conclusion

AG was an independent risk factor of inhospital all-cause mortality and was associated with adverse clinical outcomes in CCU patients. But more prospective case-control data are needed to confirm AG's role as a clinical indicator in CCU to predict prognosis.

## Figures and Tables

**Table 1:** Characteristics of patients stratified by AG quartiles.

Characteristics	Total (*n* = 3593)	Quartiles of AG (mmol/L)				*P* value
		Quartile 1 (*n* = 868) AG < 13	Quartile 2 (*n* = 902) 13 ≤ AG < 15	Quartile 3 (*n* = 779) 15 ≤ AG < 17	Quartile 4 (*n* = 1044) 15 ≤ AG < 17	
Age (years)	69.2 ± 15.0	69.6 ± 14.4	68.7 ± 15.3	69.4 ± 15.0	69.0 ± 15.1	0.679
Gender, *n* (%)						0.986
Male	2042 (56.8)	497 (57.3)	510 (56.5)	440 (56.5)	595 (57.0)	
Female	1551 (43.2)	371 (42.7)	392 (43.5)	339 (43.5)	449 (43.0)	
Race, *n* (%)						0.198
White	2551 (71.0)	640 (73.7)	641 (71.1)	554 (71.1)	716 (68.6)	
Black	263 (7.3)	52 (6.0)	66 (7.3)	53 (6.8)	92 (8.8)	
Other	779 (21.7)	176 (20.3)	195 (21.6)	172 (20.1)	236 (22.6)	
Body mass index (kg/m^2^)	28.2 ± 6.9	27.8 ± 6.7	28.3 ± 7.0	28.4 ± 6.8	28.3 ± 7.2	0.416
Diagnoses of heart diseases, *n* (%)						
Coronary artery disease	1793 (49.9)	405 (46.7)	473 (52.4)	418 (53.7)	497 (47.6)	0.006
Acute myocardial infarction	674 (18.8)	143 (16.5)	160 (17.7)	161 (20.7)	210 (20.1)	0.082
Atrial fibrillation	1349 (37.6)	319 (36.8)	334 (37.0)	304 (39.0)	392 (37.6)	0.786
Ventricular arrhythmias	206 (5.7)	31 (3.6)	39 (4.3)	47 (6.0)	89 (8.5)	<0.001
Third-degree atrioventricular block	153 (4.3)	34 (3.9)	36 (4.0)	37 (4.8)	46 (4.4)	0.820
Congestive heart failure	1935 (53.9)	421 (48.5)	461 (51.1)	415 (53.3)	638 (61.1)	<0.001
Primary cardiomyopathy	294 (8.2)	60 (6.9)	62 (6.9)	71 (9.1)	101 (9.7)	0.048
Valve disease	776 (21.6)	165 (19.0)	201 (22.3)	173 (22.2)	237 (22.7)	0.203
Endocarditis	64 (1.8)	18 (2.1)	17 (1.9)	13 (1.7)	16 (1.5)	0.824
Cardiogenic shock	471 (13.1)	70 (8.1)	92 (10.2)	94 (12.1)	215 (20.6)	<0.001
Comorbidities and medical history, *n* (%)						
Hypertension	1456 (40.5)	411 (47.4)	409 (45.3)	312 (40.1)	324 (31.0)	<0.001
Diabetes	1205 (33.5)	226 (26.0)	287 (31.8)	263 (33.8)	429 (41.1)	<0.001
Hypercholesterolemia	1200 (33.4)	313 (36.1)	314 (34.8)	271 (34.8)	302 (28.9)	0.003
Chronic lung disease	885 (24.6)	244 (28.1)	195 (21.6)	198 (25.4)	248 (23.8)	0.013
Respiratory failure	1272 (35.4)	279 (32.1)	280 (31.0)	264 (33.9)	449 (43.0)	<0.001
Chronic kidney disease	752 (20.9)	106 (12.2)	165 (18.3)	171 (22.0)	310 (29.7)	<0.001
Chronic liver disease	125 (3.5)	39 (4.5)	22 (2.4)	19 (2.4)	45 (4.3)	0.017
Malignancy	518 (14.4)	125 (14.4)	158 (17.5)	105 (13.5)	130 (12.5)	0.013
Autoimmune disease	156 (4.3)	40 (4.6)	35 (3.9)	34 (4.4)	47 (4.5)	0.879
Prior myocardial infarction	323 (9.0)	77 (8.9)	80 (8.9)	83 (10.7)	83 (8.0)	0.256
Prior stroke	85 (2.4)	19 (2.2)	24 (2.7)	12 (1.5)	30 (2.9)	0.270
Laboratory parameters						
AG (mmol/L)	15.0 ± 3.6	10.9 ± 1.2	13.5 ± 0.5	15.5 ± 0.5	19.4 ± 2.6	<0.001
Maximum AG	17.7 ± 4.3	14.7 ± 3.4	16.2 ± 2.9	17.7 ± 3.0	21.4 ± 4.1	<0.001
White blood cell (10^9^/L)	11.7 ± 5.6	10.0 ± 4.6	10.8 ± 4.8	11.7 ± 5.4	13.7 ± 6.3	<0.001
Platelet (10^9^/L)	236.8 ± 96.8	220.1 ± 91.3	234.8 ± 93.6	242.0 ± 95.1	248.4 ± 102.9	<0.001
Hemoglobin (g/dL)	11.5 ± 2.0	11.2 ± 1.8	11.4 ± 1.9	11.6 ± 2.0	11.6 ± 2.1	<0.001
Glucose (mg/dL)	154.6 ± 75.2	132.1 ± 52.0	141.5 ± 56.9	154.6 ± 69.0	184.6 ± 97.0	<0.001
Creatinine (mg/dL)	1.58 ± 1.40	1.06 ± 0.63	1.24 ± 0.79	1.45 ± 0.95	2.41 ± 2.04	<0.001
Blood nitrogen urea (mg/dL)	31.0 ± 21.6	22.6 ± 14.1	25.7 ± 16.2	31.3 ± 20.7	42.4 ± 26.2	<0.001
Sodium (mmol/L)	138.0 ± 4.6	138.4 ± 4.3	138.3 ± 4.2	138.2 ± 4.2	137.2 ± 5.3	<0.001
Potassium (mmol/L)	4.2 ± 0.8	4.0 ± 0.6	4.1 ± 0.7	4.2 ± 0.7	4.4 ± 0.9	<0.001
Medication use, *n* (%)						
Antiplatelet	2274 (63.3)	556 (64.1)	587 (65.9)	480 (61.6)	651 (62.4)	0.425
Oral anticoagulants	1047 (29.1)	259 (29.8)	276 (30.6)	232 (29.8)	280 (26.8)	0.260
Beta-blocks	2513 (69.9)	624 (71.9)	644 (71.4)	533 (68.4)	712 (68.2)	0.184
ACEI/ARB	1882 (52.4)	475 (54.7)	495 (54.9)	422 (54.2)	490 (46.9)	0.001
Statin	2095 (58.3)	512 (59.0)	552 (61.2)	458 (58.8)	573 (54.9)	0.039
Vasopressin	234 (6.5)	38 (4.4)	46 (5.1)	45 (5.8)	105(10.1)	<0.001

Continuous variables were presented as mean ± SD. Categorical variables were presented as number (percentage). Abbreviation: AG: anion gap; ACEI: angiotensin-converting enzyme inhibitor; ARB: angiotensin receptor blocker.

**Table 2:** Outcomes of patients stratified by AG quartiles.

Outcomes	Total (*n* = 3593)	Quartiles of AG (mmol/L)				*P* value
		Quartile 1 (*n* = 868) AG < 13	Quartile 2 (*n* = 902) 13 ≤ AG < 15	Quartile 3 (*n* = 779) 15 ≤ AG < 17	Quartile 4 (*n* = 1044) 15 ≤ AG < 17	
Inhospital mortality, *n* (%)	515 (14.3)	85 (9.8)	89 (9.9)	103 (13.2)	238 (22.8)	<0.001
CCU mortality, *n* (%)	399 (11.1)	55 (6.3)	73 (8.1)	88 (11.3)	183 (17.5)	<0.001
Length of CCU stay (days)	3.9 (2.8-6.6)	3.5 (2.6-5.9)	3.7 (2.8-5.9)	3.9 (2.7-6.7)	4.4 (3.0-7.3)	<0.001
Length of hospital stay (days)	8.3 (5.3-13.9)	7.3 (4.8-12.5)	7.8 (5.0-13.2)	8.5 (5.3-13.7)	9.7 (4.0-15.7)	<0.001
Acute kidney injury, *n* (%)	1924 (53.6)	371 (42.7)	434 (48.1)	429 (55.1)	690 (66.1)	<0.001
Maximum SOFA	4 (2-6)	3 (1-5)	3 (2-5)	4 (2-6)	5 (3-8)	<0.001
Maximum SAPSII	36 (28-46)	33 (27-40)	34 (26-43)	36 (28-44)	42 (33-52)	<0.001

Nonnormally distributed continuous variables were presented as median (IQR). Categorical variables were presented as number (percentage). Abbreviation: AG: anion gap; CCU: coronary care unit; SOFA: sequential organ failure assessment score; SAPS II: simplified acute physiology score II.

**Table 3:** The association between AG and inhospital all-cause mortality.

	AG (mmol)		
	OR (95% CI)	*P* value	*P* for trend
Model 1			<0.001
Quartile 1: AG < 13	Ref		
Quartile 2: 13 ≤ AG < 15	1.01 (0.74-1.38)	0.958	
Quartile 3: 15 ≤ AG < 17	1.40 (1.03-1.90)	0.029	
Quartile 4: AG ≥ 17	2.72 (2.08-3.55)	<0.001	
Continuous	1.13 (1.10-1.16)	<0.001	
Model 2			<0.001
Quartile 1: AG < 13	Ref		
Quartile 2: 13 ≤ AG < 15	1.02 (0.75-1.40)	0.891	
Quartile 3: 15 ≤ AG < 17	1.40 (1.03-1.91)	0.031	
Quartile 4: AG ≥ 17	2.78 (2.12-3.63)	<0.001	
Continuous	1.14 (1.11-1.16)	<0.001	
Model 3			
Quartile 1: AG < 13	Ref		<0.001
Quartile 2: 13 ≤ AG < 15	1.02 (0.72-1.45)	0.897	
Quartile 3: 15 ≤ AG < 17	1.22 (0.86-1.73)	0.258	
Quartile 4: AG ≥ 17	1.49 (1.07-2.09)	0.019	
Continuous	1.06 (1.02-1.09)	0.001	

Models were derived from binary logistic regression analysis. Model 1: unadjusted. Model 2: adjusted for age, gender, and race. Model 3: adjusted for age, gender, race, body mass index, coronary heart disease, acute myocardial infarction, atrial fibrillation, ventricular arrhythmias, third-degree atrioventricular block, congestive heart failure, primary cardiomyopathy, valve disease, endocarditis, cardiogenic shock, hypertension, diabetes, hypercholesterolemia, respiratory failure, chronic kidney disease, chronic liver disease, chronic lung disease, malignancy, autoimmune disease, prior myocardial infarction, prior stroke, oral anticoagulants, statin, vasopressin, angiotensin-converting enzyme inhibitor, angiotensin receptor blocker, antiplatelet, blood nitrogen urea, white blood cell, sodium, and creatinine. Abbreviation: AG: anion gap; OR: odds ratio; CI: confidence interval.

**Table 4:** Subgroup analysis of associations between inhospital all-cause mortality and AG (mmol/L).

Subgroups	*n*	Quartile 1 AG < 13	Quartile 2 13 ≤ AG < 15	Quartile 3 15 ≤ AG < 17	Quartile 4 15 ≤ AG < 17	*P* for interaction
Gender						0.839
Male	2042	Ref	1.21 (0.80-1.84)	1.32 (0.86-2.01)	2.98 (2.07-4.27)	
Female	1551	Ref	0.79 (0.49-1.27)	1.50 (0.96-2.33)	2.43 (1.64-3.61)	
Age (years)						0.968
<72	1832	Ref	1.08 (0.67-1.75)	1.09 (0.66-1.80)	2.82 (1.87-4.25)	
≥72	1761	Ref	0.97 (0.64-1.47)	1.62 (1.10-2.39)	2.71 (1.90-3.85)	
Race						0.244
White	2551	Ref	1.07 (0.74-1.54)	1.30 (0.91-1.87)	2.57 (1.88-3.52)	
Black	263	Ref	0.14 (0.16-1.28)	0.77 (0.19-3.03)	2.29 (0.80-6.57)	
Other	779	Ref	1.13 (0.57-2.22)	1.98 (1.05-3.73)	3.41 (1.91-6.07)	
Body mass index (kg/m^2^)						0.332
<27	1792	Ref	0.96 (0.64-1.46)	1.49 (0.99-2.22)	2.45 (1.72-3.48)	
≥27	1801	Ref	1.08 (0.67-1.75)	1.35 (0.84-2.17)	3.14 (2.09-4.72)	
Coronary artery disease						0.530
Yes	1793	Ref	1.20 (0.73-1.97)	1.68 (1.04-2.73)	3.10 (2.00-4.81)	
No	1800	Ref	0.94 (0.62-1.41)	1.31 (0.88-1.95)	2.55 (1.82-3.57)	
Acute myocardial infarction						0.269
Yes	674	Ref	1.15 (0.50-2.62)	2.10 (0.99-4.46)	3.65 (1.83-7.30)	
No	2919	Ref	0.99 (0.70-1.39)	1.29 (0.92-1.81)	2.58 (1.93-3.45)	
Atrial fibrillation						0.921
Yes	1349	Ref	1.16 (0.72-1.86)	1.40 (0.88-2.24)	2.91 (1.93-4.40)	
No	2244	Ref	0.90 (0.59-1.37)	1.39 (0.93-1.08)	2.59 (1.83-3.68)	
Ventricular arrhythmias						0.065
Yes	206	Ref	6.56 (0.76-56.55)	9.17 (1.12-75.13)	17.68 (2.30-135.7)	
No	3387	Ref	0.94 (0.68-1.30)	1.29 (0.94-1.76)	2.45 (1.86-3.22)	
Third-degree atrioventricular block						0.687
Yes	153	Ref	0.30 (0.03-2.99)	0.91 (0.17-4.86)	2.51 (0.63-10.1)	
No	3440	Ref	1.04 (0.76-1.42)	1.43 (1.05-1.95)	2.73 (2.08-3.58)	
Congestive heart failure						0.013
Yes	1935	Ref	0.84 (0.56-1.27)	1.15 (0.77-1.71)	1.97 (1.40-2.78)	
No	1658	Ref	1.26 (0.77-2.05)	1.78 (1.11-2.88)	4.07 (2.66-6.23)	
Primary cardiomyopathy						0.994
Yes	294	Ref	0.97 (0.23-4.05)	1.78 (0.51-6.22)	2.64 (0.84-8.29)	
No	3299	Ref	1.01 (0.73-1.39)	1.39 (1.01-1.91)	2.76 (2.10-3.64)	
Valve disease						0.919
Yes	776	Ref	0.75 (0.35-1.60)	1.38 (0.69-2.78)	2.34 (1.264.37)	
No	2817	Ref	1.08 (0.77-1.53)	1.42 (1.01-1.99)	2.84 (2.12-3.82)	
Endocarditis						0.617
Yes	64	Ref	0.67 (0.10-4.58)	0.91 (0.13-6.40)	3.89 (0.80-18.97)	
No	3529	Ref	1.02 (0.74-1.40)	1.42 (1.04-1.94)	2.71 (2.07-3.56)	
Cardiogenic shock						0.352
Yes	471	Ref	1.00 (0.48-2.09)	1.43 (0.70-2.92)	1.88 (1.01-3.51)	
No	3122	Ref	0.97 (0.68-1.37)	1.30 (0.92-1.83)	2.55 (1.88-3.44)	
Hypertension						0.126
Yes	1456	Ref	1.01 (0.60-1.67)	1.50 (0.90-2.48)	3.44 (2.21-5.38)	
No	2137	Ref	1.00 (0.67-1.48)	1.30 (0.88-1.91)	2.27 (1.62-3.17)	
Diabetes						0.914
Yes	1205	Ref	1.35 (0.73-2.49)	1.72 (0.94-3.13)	2.94 (1.72-5.02)	
No	2388	Ref	0.91 (0.63-1.32)	1.32 (0.93-1.90)	2.79 (2.04-3.82)	
Hypercholesterolemia						0.019
Yes	1200	Ref	1.54 (0.80-2.95)	2.22 (1.18-4.19)	4.89 (2.75-8.69)	
No	2393	Ref	0.88 (0.61-1.26)	1.20 (0.84-1.71)	2.17 (1.60-2.95)	
Chronic lung disease						<0.001
Yes	885	Ref	0.94 (0.56-1.56)	0.72 (0.42-1.22)	1.55 (0.99-2.41)	
No	2708	Ref	1.18 (0.78-1.77)	2.04 (1.39-3.01)	3.82 (2.70-5.42)	
Respiratory failure						0.005
Yes	1272	Ref	0.71 (0.46-1.09)	1.14 (0.76-1.72)	1.67 (1.18-2.38)	
No	2321	Ref	1.62 (0.99-2.65)	1.85 (1.12-3.04)	4.26 (2.74-6.62)	
Chronic kidney disease						0.813
Yes	752	Ref	1.49 (0.65-3.40)	1.35 (0.59-3.10)	2.86 (1.37-5.97)	
No	2841	Ref	0.93 (0.66-1.31)	1.45 (1.04-2.01)	2.78 (2.08-3.73)	
Chronic liver disease						0.538
Yes	125	Ref	1.22 (0.30-4.90)	1.03 (0.23-4.66)	2.23 (0.76-6.60)	
No	3468	Ref	1.02 (0.74-1.40)	1.44 (1.05-1.97)	2.76 (2.10-3.63)	
Malignancy						0.109
Yes	518	Ref	0.97 (0.49-1.94)	1.14 (0.55-2.39)	1.82 (0.95-3.52)	
No	3075	Ref	1.00 (0.70-1.42)	1.47 (1.05-2.06)	2.94 (2.19-3.95)	
Autoimmune disease						0.680
Yes	156	Ref	0.53 (0.12-2.30)	1.21 (0.35-4.18)	1.94 (0.65-5.77)	
No	3437	Ref	1.04 (0.76-1.44)	1.42 (1.04-1.94)	2.78 (2.11-3.66)	
Prior myocardial infarction						0.706
Yes	323	Ref	1.43 (0.51-3.97)	1.22 (0.43-3.44)	2.77 (1.09-7.06)	
No	3270	Ref	0.97 (0.70-1.35)	1.43 (1.04-1.96)	2.71 (2.06-3.58)	
Prior stroke						0.413
Yes	61	Ref	-	3.6 (0.29-44.82)	3.6 (0.39-33.50)	
No	3508	Ref	1.03 (0.75-1.41)	1.38 (1.02-1.88)	2.72 (2.08-3.55)	
Antiplatelet						0.678
Yes	2274	Ref	1.02 (0.69-1.53)	1.61 (1.09-2.36)	2.81 (2.00-3.96)	
No	1319	Ref	0.99 (0.60-1.64)	1.12 (0.68-1.84)	2.57 (1.68-3.93)	
Oral anticoagulants						0.011
Yes	1047	Ref	0.99 (0.51-1.89)	0.57 (0.26-1.25)	1.57 (0.86-2.86)	
No	2546	Ref	1.02 (0.71-1.46)	1.69 (1.20-2.37)	3.06 (2.26-4.13)	
Beta-blockers						0.002
Yes	2513	Ref	0.90 (0.61-1.31)	1.24 (0.85-1.80)	1.98 (1.43-2.76)	
No	1080	Ref	1.28 (0.74-2.23)	1.75 (1.03-2.98)	4.58 (2.86-7.34)	
ACEI/ARB						<0.001
Yes	1882	Ref	0.96 (0.58-1.59)	1.17 (0.71-1.95)	1.40 (0.87-2.24)	
No	1711	Ref	1.04 (0.70-1.56)	1.56 (1.06-2.31)	3.43 (2.45-4.81)	
Statin						0.846
Yes	2095	Ref	1.02 (0.65-1.60)	1.45 (0.93-2.24)	2.62 (1.78-3.85)	
No	1498	Ref	1.02 (0.66-1.59)	1.37 (0.89-2.10)	2.75 (1.90-3.98)	
Vasopressin						0.042
Yes	234	Ref	0.65 (0.27-1.56)	0.89 (0.37-2.11)	1.18 (0.56-2.47)	
No	3359	Ref	1.05 (0.74-1.48)	1.45 (1.04-2.04)	2.78 (2.06-3.74)	
White blood cell (10^9^/L)						0.057
<10.5	1778	Ref	0.80 (0.52-1.23)	1.22 (0.79-1.87)	1.91 (1.29-2.84)	
≥10.5	1815	Ref	1.25 (0.78-2.01)	1.54 (0.98-2.43)	3.10 (2.07-4.65)	
Platelet (10^9^/L)						0.170
<221	1793	Ref	1.10 (0.71-1.72)	1.80 (1.18-2.75)	3.30 (2.27-4.81)	
≥221	1800	Ref	0.91 (0.58-1.41)	1.07 (0.69-1.66)	2.22 (1.52-3.24)	
Hemoglobin (g/dL)						0.223
<11.4	1777	Ref	0.93 (0.62-1.39)	1.24 (0.82-1.86)	2.42 (1.70-3.43)	
≥11.4	1816	Ref	1.16 (0.71-1.90)	1.73 (1.09-2.76)	3.31 (2.17-5.04)	
Glucose (mg/dL)						0.828
<132	1773	Ref	1.12 (0.74-1.70)	1.42 (0.93-2.16)	2.88 (1.97-4.22)	
≥132	1820	Ref	0.86 (0.53-1.39)	1.32 (0.84-2.08)	2.44 (1.65-3.62)	
Creatinine (mg/dL)						0.177
<1.1	1537	Ref	0.99 (0.64-1.54)	1.24 (0.78-1.97)	2.20 (1.40-3.45)	
≥1.1	2056	Ref	0.96 (0.61-1.52)	1.37 (0.89-2.11)	2.52 (1.72-3.68)	
Blood nitrogen urea (mg/dL)						0.365
<24	1728	Ref	1.08 (0.68-1.73)	1.34 (0.81-2.20)	2.79 (1.77-4.39)	
≥24	1865	Ref	0.83 (0.54-1.27)	1.06 (0.71-1.27)	1.76 (1.24-2.50)	
Sodium (mmol/L)						0.123
<138	1456	Ref	1.16 (0.73-1.84)	1.26 (0.79-2.00)	2.28 (1.53-3.39)	
≥138	2137	Ref	0.89 (0.58-1.37)	1.50 (1.00-2.25)	3.02 (2.10-4.33)	
Potassium (mmol/L)						0.290
<4.1	1625	Ref	0.95 (0.60-1.50)	1.70 (1.09-2.64)	3.12 (2.09-4.65)	
≥4.1	1968	Ref	1.04 (0.68-1.61)	1.17 (0.77-1.79)	2.39 (1.66-3.44)	

Binary logistic regression analysis was used, and results were presented as OR (odds ratio) and 95% CI (confidence interval). Abbreviation: AG: anion gap; ACEI: angiotensin-converting enzyme inhibitor; ARB: angiotensin receptor blocker.

## Data Availability

All data used in this analysis were from an openly available critical care database named MIMIC-III. Protecting Human Research Participant exam was passed to gain access to MIMIC-III database, and our certificate number is 9027152.
